# Peptide halogenation biochemistry: interfacing pharmaceutical deliverables with chemical innovation

**DOI:** 10.1007/s00044-025-03488-0

**Published:** 2025-10-23

**Authors:** Vinayak Agarwal

**Affiliations:** 1 School of Chemistry and Biochemistry, Georgia Institute of Technology, Atlanta, GA USA; 2 School of Biological Sciences, Georgia Institute of Technology, Atlanta, GA USA

**Keywords:** Peptide, Natural product, Halogenase, Biosynthesis, Antibiotic

## Abstract

The biosynthetic schemes for the production of halogenated peptidic natural products offer avenues for the discovery of peptide halogenases, and opportunities for development of biocatalysts for derivatization of peptides and proteins. Here, a short review of recent discoveries regarding biocatalytic protein and peptide halogenation is provided. Halogenation in two major classes of peptidic natural products is discussed, those that are produced as ribosomal peptides and post translationally modified, and those that are produced by assembly line-like non ribosomal peptide synthetases. Mechanistic considerations and biocatalytic applications of peptide halogenases are briefly discussed.

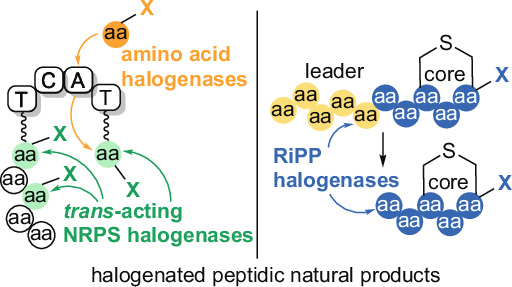

Peptides and peptidic natural products are often endowed with favorable pharmacological bioactivities. Popular examples include the hormone insulin, antibiotics vancomycin and daptomycin, venomous peptides that are adapted for treatment of chronic pain, and glucagon-like peptide-1 (GLP-1) agonists that are used for the management of type II diabetes. Peptidic natural products are typically sourced by post translational modifications affected upon ribosomally synthesized peptides or as peptides that are assembled by megadalton assembly line-like non ribosomal peptide synthetases (NRPSs) [1, 2]. Peptidic natural products are principally divided among the ribosomally derived and post translationally derived peptides (RiPPs) and NRPS-derived peptides (NRPs) modalities. In addition to peptidic natural products, the prominence of synthetic peptides in pharmacology is well established as exemplified by the recent surge of interest in designer, non-natural peptides in modulating biomolecular interactions [3–5]. The clinical deliverables achieved by peptidic pharmacophores are juxtaposed against their chemical complexity and avenues for chemical innovation that allow for structural diversification, bioactivity tailoring, modulation of stability and transport phenomenon, and pharmacokinetic properties.

Halogenation is often a bioactivity conferring modification for polyketide and alkaloid natural products [6, 7]. This assertion extends to peptidic natural products as well. Among RiPPs, brominated darobactin analogs demonstrated higher antibiotic efficacy against Gram-negative bacterial pathogens and tryptophan halogenation likewise increased the antimicrobial potency of the lanthipeptide NAI-107 [8–10]. Among NRPs, the dependence of antibiotic efficacy of vancomycin on phenyl chlorination is well established and for dipeptidic natural products, the iodination status of the thyroid hormone determines its biological activity [11–13]. Without a doubt, halogenated intermediates also underlie the chemical synthesis of peptidic molecules particularly as it relates to inter- and intramolecular cross coupling of amino acid side chains (Fig. 1) [14–17]. Motivated by the biological activity of halogenated peptidic natural products and the highly desirable chemoenzymatic access to halogenated peptidic intermediates, the activity of halogenating enzymes—halogenases—is gathering contemporary interest [18–21].

**Fig. 1:**
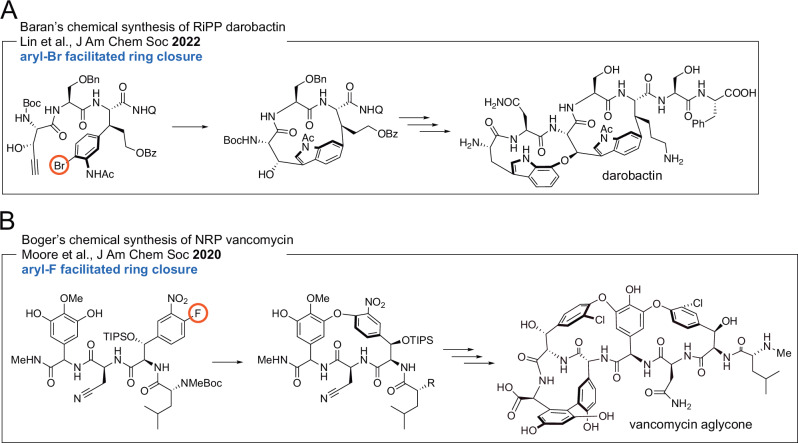
Illustrative applications of aryl halides in the chemical syntheses of peptidic natural products. Both examples, (**A**) Baran’s synthesis of the RiPP darobactin (also note Sarlah’s synthesis of darobactin [15]), and (**B**) Boger’s synthesis of the NRP vancomycin both use halogenation as a C–H activation strategy to catalyze peptide macrocyclization

The enzymatic basis for chlorination in NRP glycopeptide antibiotic biosynthesis has attracted attention; biogenic incorporation of fluorinated amino acids in glycopeptide antibiotics has also been realized [22]. In 2014, Robinson and co-workers reconstituted the activity of the flavin-dependent halogenase (FDH) VhaA that is responsible for the incorporation of both chlorine atoms in vancomycin. The authors found that the requisite substrate for VhaA was a NRPS assembly line hexapeptidic intermediate (Fig. 2A) [23]. Curiously, the activity of the halogenase was dependent on Cβ-hydroxylation of the L-tyrosine residues that were chlorinated by VhaA. These tyrosine residues were the second and the sixth residues to be incorporated by the NRPS assembly line. Two other electron rich side chains of D-hydroxyphenylglycine residues in the hexapeptidic substrate were not chlorinated by VhaA. While the structural insights are unavailable, these observations denote highly specific substrate binding in the VhaA active site. Given these constraints, the biotechnological potential of VhaA for chlorination of other glycopeptide antibiotic scaffolds would conceivably be rather low.

**Fig. 2:**
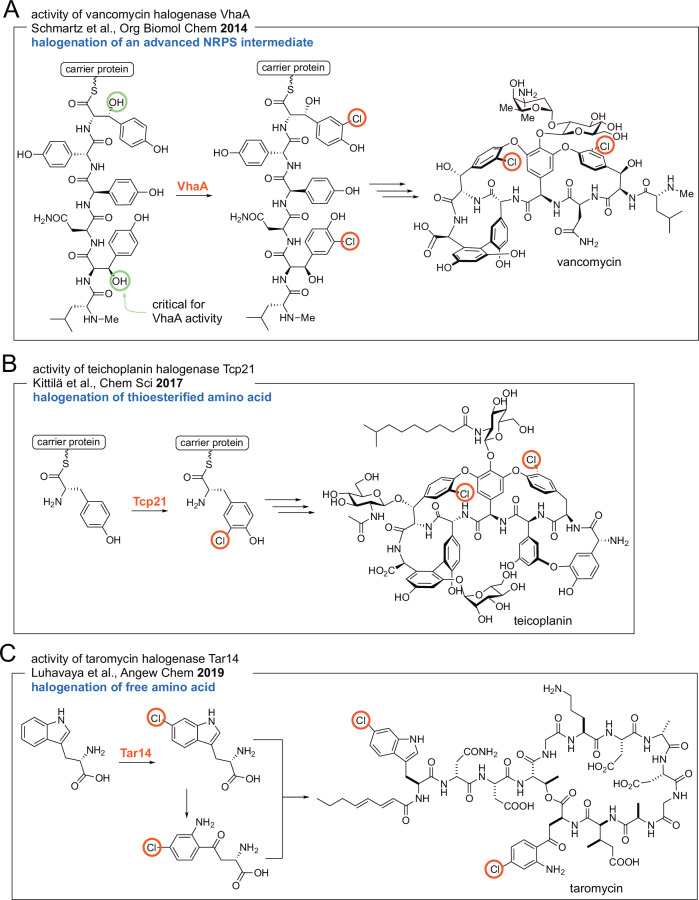
Different timing of halogenation events during NRP biosynthesis. **A** The halogenase VhaA dichlorinates a thioesterified hexapeptide that is further extended and oxidatively tailored to yield vancomycin. Note that in the absence of tyrosine Cβ-hydroxylation (residues 2 and 6 of the hexapeptide substrate), halogenation by VhaA did not proceed. **B** The halogenase Tcp21 chlorinates a thioesterified tyrosine. The chlorinated tyrosine would then be extended by the NRPS assembly line to furnish teicoplanin. **C** Tar14 chlorinates free tryptophan, that is then converted to chlorinated kynurenine. Chlorinated tryptophan and chlorinated kynurenine are then incorporated into the NRP

Shortly after, Stegmann, Cryle, and coworkers reported findings regarding the halogenation of glycopeptide antibiotics balhimycin and teicoplanin. Unlike the vancomycin halogenase VhaA for which a hexapeptidic intermediate was identified as the substrate, the teicoplanin halogenase Tcp21 demonstrated activity only for carrier protein thioesterified L-tyrosine; activity was not observed for di-, hexa-, and heptapeptidic intermediates for the NRPS assembly line (Fig. 2B) [24]. This finding was supplemented by genomic reconstruction of the balhimycin NRPS assembly line to offload a halogenated dipeptidic intermediate which offered support to the hypothesis that halogenation occurs upon the thioesterified tyrosine prior to condensation with the upstream peptidic intermediate [24]. In contrast to vancomycin biosynthesis where halogenation occurred after peptide assembly, substrate selectivity of the teicoplanin and balhimycin NRPS condensation domains would likely dictate that only halogenated tyrosine would proceed along the peptide assembly line for further elongation. Similar gatekeeping selectivity for halogenated building blocks has been established for ketosynthase domains in polyketide synthases [25]. The same scenario presents itself during chlorination of the thioesterified piperazate residue in kutznerides by the nonheme Fe(II)-dependent halogenase KthP [26]. A second nonheme Fe(II)-dependent halogenase KtzD chlorinates a thioesterified L-isoleucine side chain in a cryptic manner to set up the installation of the cyclopropyl ring in the allocoronamic acid building block [27]. Cryptic halogenation finds widespread application in natural product biosynthesis [28].

Regardless of the exact timing of the enzymatic modification, halogenation of a thioesterified amino acid building block or that of a thioesterified peptidic intermediate can both be envisaged within context of *trans*-acting halogenases that interface with the NRPS assembly line; the interaction of *trans*-acting modification enzymes with NRPS assembly lines is an area of active investigation particularly as it relates to the oxidative biaryl couplings in glycopeptide antibiotic biosynthesis [29, 30]. An entirely different scenario presents itself during the biosynthesis of the NRP taromycin [31]. Moore and co-workers reported that the halogenase Tar14 chlorinated free L-tryptophan that was then incorporated in the NRP. Furthermore, L-6-chlorotryptophan generated by Tar14 was enzymatically morphed into L-4-chlorokynurenine which was also incorporated into the NRP (Fig. 2C) [32]. Here, halogenation proceeds on the free amino acid building prior to it being loaded onto the NRPS carrier proteins. Thus, the selectivity of the adenylation domains in the Tar NRPS would likely dictate that only the halogenated building blocks are incorporated into the NRP. Unlike KthP and KthD mentioned above, dichlorination en route kutzneride biosynthesis is also affected upon free tryptophan by the FDHs KtzQ and KtzR, followed by incorporation of 6,7-dichlorotryptophan in the NRPS assembly line [33]. Recently, the flavoenzymes JasF and KrmI have been implicated in the production of the bromotryptophan containing macrocyclic marine NRPs jaspamide/jasplakinolide and keramamides, respectively [34, 35]. While the activity of JasF remains to be reconstituted, the domain architecture of JasF resembles that of FAD-containing NAD(P)H-dependent oxidoreductases reminiscent of single component marine tryptophan brominases Bmp5 and AetF wherein the flavin reductase and halide oxidase domains are fused into a single polypeptide [36, 37]. Canonical FDHs such as Tar14, KtzQ, and KtzR mentioned refer only to the halide oxidases with the flavin reductase participating in the halogenation reaction as a separate partner protein; the flavin reductase may or may not be encoded in the same biosynthetic gene cluster as the halogenase. The reader is directed to excellent reviews describing the reaction mechanism of FDHs though it should be noted that several mechanistic details regarding the halogenase catalytic cycle in FDHs are still being worked out [38–41].

Enzymatic halogenation in RiPP biosynthesis has also been realized. Contrary to the two models of halogenation in NRP biosynthesis presented above where halogenation occurs either on free amino acids or en route peptide assembly, chlorination during the biosynthesis of the RiPP antibiotic NAI-107 occurs after all other modifications on the RiPP precursor peptide have been installed, and the post-translationally modified C-terminal core has been proteolytically removed from the unmodified N-terminal core peptide [1, 42]. Nair and van der Donk have described the activity and structure of the FDH MibH to reveal a highly specific catalyst the activity of which was dependent on the peptide macrocyclization as well as proline side chain hydroxylation (Fig. 3A) [42]. Note the unique substrate profile for MibH; while it is highly specific for modifications in the core, the leader peptide had to be proteolytically removed to yield a viable substrate.

**Fig. 3:**
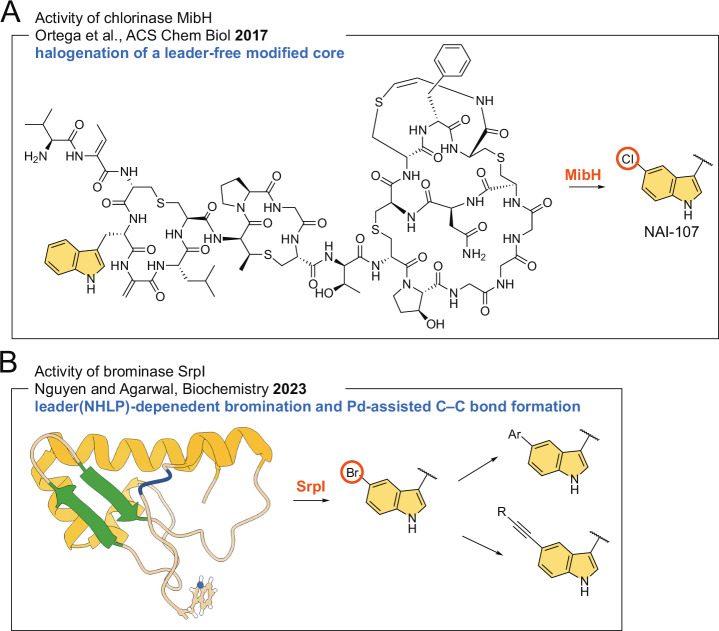
Halogenation in RiPP biosynthesis. **A** Tryptophan chlorination for biosynthesis of NAI-107 catalyzed by MibH. Note that MibH chlorinates the tryptophan side chain indole when other modification such as macrocyclization and proline side chain hydroxylation have occurred, and the leader peptide has been removed. **B** Halogenase SrpI brominates the C-terminal tryptophan side chain in a leader-dependent fashion. The structure of the NHLP is shown in cartoon representation with the boundary between the leader and the core regions denoted in blue. The brominated indole can be further modified using Suzuki-Miyaura and Sonogashira coupling reactions

MibH served as a genetic hook to mine metagenomes for the detection of RiPP biosynthetic gene loci encoding the FDH SrpI [43]. Biochemical investigations revealed that SrpI was an obligate brominase—it did not possess any chlorination activity [43]. Unlike MibH, the activity of SrpI required the presence of the leader sequence in the precursor peptide SrpE [44]. SrpI possessed moderate substrate selectivity, in that, it regiospecifically brominated C-terminal tryptophan side chains for penta- to heptapeptides (Fig. 3B) [45]. However, the requirement of the long nitrile hydratase-like leader peptide (NHLP) compromised the atom economy for the delivery of a brominated peptidic product [46]. Detailed spectroscopic experiments identified a drastically reduced SrpE leader sequence that was sufficient to support SrpI activity [47]. Peptide bromination was found to be particularly useful in the identification of transition metal-assisted cross-coupling reaction conditions for peptide and protein diversification which include C–C bond formation using Suzuki-Miyaura and Sonogashira coupling reactions [44, 45]. Further C–C and C–N bond forming reactions that have been developed for small molecule aryl bromides can similarly be adapted for halogenated peptides and proteins with rigourous screening of catalysts and reaction conditions. While SrpI modified linear peptides, the FDH MppI was identified to brominate a macrocyclic lanthipeptide in a leader-dependent fashion [48]. At the moment, enzyme reaction mechanisms for the FDHs that halogenate peptidic substrates are thought to mirror those that halogenate smaller non-peptidic substrates and amino acids. As such, key active site residues have been experimentally demonstrated to be conserved between FDHs that halogenate peptidic and non-peptidic substrates [43, 48, 49]. Furthermore, these residues are conserved between chlorinases, as well as brominases. Enzymatic chlorination and bromination are expected to proceed using identical mechanistic routes, though the redox potential of the flavin cofactor could determine the identity of the most electronegative halide that the FDH could incorporate into the organic co-substrate.

Recently, a bioinformatic search by Mitchell and coworkers identified chlorinated lasso peptide RiPPs [50]. Biochemical characterization of the lasso peptide chlorinase by Luo, Dong, and coworkers identified the chlorinase ChlH to be chlorinating two of the three tryptophan side chains in the RiPP chlorolassin [51]. Consistent with glycopeptide NRPs and the RiPP NAI-107, dichlorination enhanced the antibacterial of the lasso peptide as well. All above-mentioned enzymes are FDHs [40]. It should be noted that the enzymology of FDHs is rooted in the halogenation of free tryptophan, and that various tryptophan halogenases and their engineered variants find utility in the halogenation of peptides and proteins [52, 53].

Noteworthy here is the prominence of FDHs in the production of halogenated peptidic natural products, though isolated examples of nonheme Fe(II)-dependent halogenases involved in the production of kutznerides are described above. Among other mechanistic classes of halogenases, vanadium-dependent haloperoxidases (VHPOs) find applications as general-purpose halogenation biocatalysts due to their expanded substrate scope with Biegasiewicz and Gulder, among others, providing recent examples of their utility [54–58]. As for FDHs, mechanistic details for the VHPO catalytic cycle are under investigation [59–61]. VHPOs and FDHs both catalyze halide to halonium oxidation with ongoing debate regarding the chemical nature and degree of enzyme-exercised control over the reactive halonium species in aqueous media. Regardless, VHPOs are often found to be substrate promiscuous with the inherent reactivity of the substrate directing the site for electrophilic halogenation [40]. On the other hand, FDHs tend to be more substrate selective. This selectivity can be leveraged for regiospecific halogenation outcomes and combined with engineering and evolution-based strategies for expanding their substrate scope [62]. Interest in biocatalytic halogenation of peptides and proteins sustained considering the medicinal relevance of halogenated metabolites and opportunities for chemical derivatization that the C–(halogen) bond provides. Enzymatic halogenation is complementary to other synthetic biology techniques, such as genetic code expansion and leveraging reactivity of amino acid side chains for the installation of halogen handles in peptides and proteins [63, 64].

Cyclic peptides, and macrocyclized peptide natural products are considered to be the Goldilocks modalities that bridge the membrane permeation and bioavailability characteristics of small molecules with the specifity and engagement to large biomolecular surfaces of biologics [65–67]. As such, halogenation can improve upon the bioactivities of these molecules as is discussed for exemplifying NRPs and RiPPs discussed in the text above. More importantly, peptide halogenation provides a bioorthogonal handle for further derivatization of peptides and proteins. While preliminary forays have been made in this space, much more progress remains to be realized. Also equally exciting is the ability of halogenase encoding genes to act as beacons to guide genome mining for the discovery of halogenated NRPs and halogenated RiPPs. As such, the prominence of peptidic halogenation biochemistry in medicinal chemistry and in biotechnology is envisaged to grow and magnify in the near future.

## Data Availability

No datasets were generated or analysed during the current study.
